# Physically Active Lifestyle Does Not Decrease the Risk of Fattening

**DOI:** 10.1371/journal.pone.0004745

**Published:** 2009-03-09

**Authors:** Klaas R. Westerterp, Guy Plasqui

**Affiliations:** Department of Human Biology, Maastricht University, Maastricht, The Netherlands; Karolinska Institutet, Sweden

## Abstract

**Background:**

Increasing age is associated with declining physical activity and a gain in fat mass. The objective was to observe the consequence of the age-associated reduction in physical activity for the maintenance of energy balance as reflected in the fat store of the body.

**Methodology/Principal Findings:**

Young adults were observed over an average time interval of more than 10 years. Physical activity was measured over two-week periods with doubly labeled water and doubly labeled water validated triaxial accelerometers, and body fat gain was measured with isotope dilution. There was a significant association between the change in physical activity and the change in body fat, where a high initial activity level was predictive for a higher fat gain.

**Conclusion/Significance:**

The change from a physically active to a more sedentary routine does not induce an equivalent reduction of energy intake and requires cognitive restriction to maintain energy balance.

## Introduction

Doubly labeled water studies show that physical activity induced energy expenditure decreases on average with more than 50% between the age of 20–30 and over 65 [1]. Exercise training does not seem to prevent the age-associated decline in physical activity because of compensation by a decrease in non-training physical activity [1]. Thus, a physically active lifestyle inevitably results in a larger decrease of daily energy expenditure at later age than a sedentary lifestyle. Here, longitudinal measurements were performed to observe the consequence of the age-associated reduction in physical activity for the maintenance of energy balance as reflected in the fat store of the body.

## Methods

Healthy, non-obese adults (17 women and 23 men; 27±5 yr; 22.8±2.0 kg m^−2^) participated in the study. All subjects gave written informed consent to participate in the study, which was approved by the Ethics Committee of the Maastricht University Medical Centre. Follow-up measurements were performed after 11±4 (range 6–16) yr. Total energy expenditure was measured by monitoring their metabolism with doubly labeled water over a two-week period, the optimal observation interval for the biological half-lives of the isotopes [2]. The physical activity level (PAL) is defined as the factor by which total energy expenditure exceeds resting energy expenditure, measured in the early morning with indirect calorimetry. All initial measurements and follow-up measurements in 22 subjects were performed with doubly labeled water. Additionally, all follow-up measurements of PAL, including the remaining 18 subjects, were performed over the same period with a doubly labeled water validated triaxial accelerometer [3]. Body fat was assessed with deuterium dilution.

## Results

**Figure 1:**
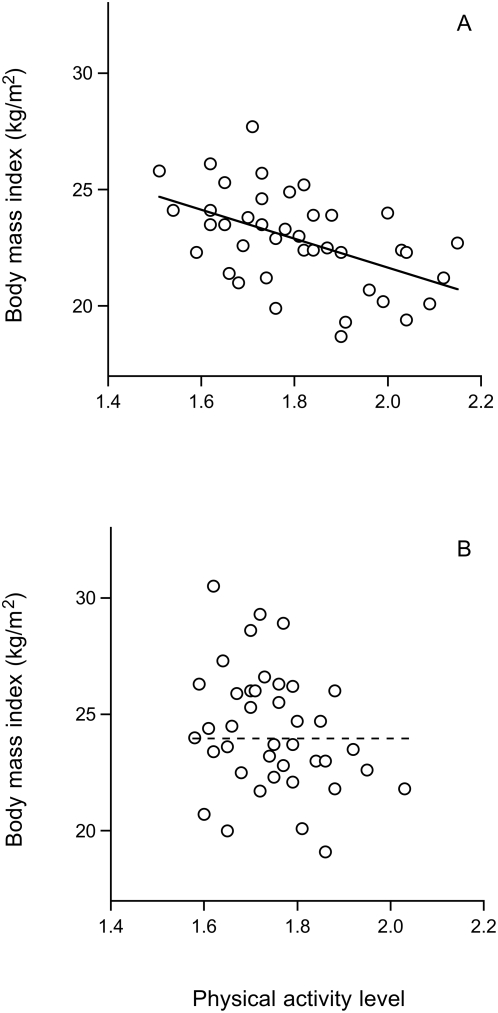
Body mass index as a function of the physical activity level at baseline (A) and at follow up (B). Linear regression analysis shows an inverse relationship at baseline (A, continuous line) and no relationship at follow-up (B, discontinuous line), 11±4 yr later, in 40 subjects.

**Figure 2:**
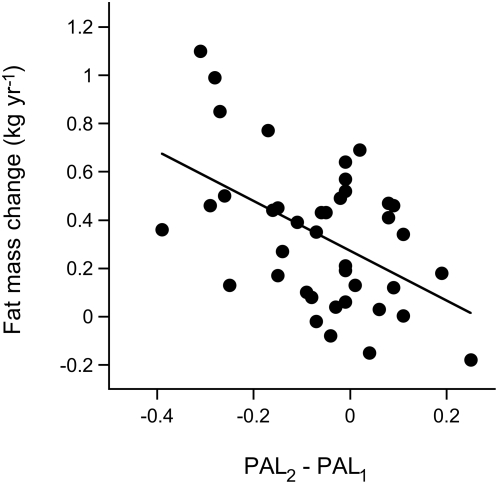
Change in fat mass as a function of the difference in physical activity level. Physical activity level at baseline (PAL_1_) and at follow up (PAL_2_), 11±4 yr later, in 40 healthy subjects with body-mass indices within the normal range. Linear regression shows an inverse relationship between the change in PAL and the rate of fat mass change.

Body mass index, as a general indicator of body fatness, increased from 22.8±2.0 kg/m^2^ at baseline to 24.3±2.6 kg/m^2^ at follow-up (P<0.01, Table 1). Total energy expenditure showed a non-significant decrease and resting energy expenditure showed a non-significant increase, in combination resulting in a significant decrease of activity energy expenditure from 4.21±1.05 MJ/d to 3.92±1.19 MJ/d (P<0.05). The PAL value decreased significantly (P<0.01) from 1.81±0.16 (range 1.51–2.15) to 1.75±0.11 (range 1.58–2.03). A similar reduction (P<0.05), from 1.84±0.17 (range 1.51–2.15) to 1.74±0.16 (range 1.43–2.08), was observed in the subgroup of 22 subjects where both PAL measurements were performed with doubly labeled water. The decrease in PAL was related to the initial PAL value (r^2^ = 0.60, P<0.0001), where physically active subjects showed a larger reduction. At baseline, body mass index was negatively related to PAL (r^2^ = 0.25, P<0.001; Figure 1A), while at follow-up the relation had disappeared. (Figure 1B). The majority of the subjects showed an increase in body fat with a mean value of 0.34±0.30 kg yr^−1^ (P<0.0001). The rate of change of fat mass was positively related with baseline PAL (P<0.05) and inversely related to the difference in PAL between baseline and follow-up (P = 0.01; Figure 2). Thus, subjects with a higher PAL at baseline gained more fat.

**Table 1:** Subject characteristics and energy expenditure at baseline and follow-up.

	Baseline	Follow-up
Age (y)	27±5	39±8***
Body mass index (kg/m^2^)	22.8±2.0	24.3±2.6**
Resting energy expenditure (REE, MJ/d)	6.76±0.98	6.84±1.00
Toal energy expenditure (TEE, MJ/d)	12.19±1.82	11.95±1.77
Activity energy expenditure (0.9TEE-REE, MJ/d)1)	4.21±1.05	3.92±1.19*
Physical activity level (TEE/REE)	1.81±0.16	1.75±0.11**

1) Calculation based on a fixed 10% of TEE for diet induced energy expenditure.

* P<0.05; ** P<0.01; *** P<0.001 for difference with baseline (n = 40).

## Discussion

The PAL of the subjects was typical for the general population, where doubly labeled water assessed PAL ranges between 1.5 and 2.1 for sedentary and very active people [4]. The earlier reported age associated reduction of PAL and the gain in fat mass was deducted from cross-sectional data, comparing young adults with elderly, and the two phenomena could not be linked together [1]. The current analysis shows a significant gain in fat mass in adults already from the age of 27±5 yr onwards, observed again after 11±4 (range 6–16) yr, and a close link with a change in PAL. Fat mass change ranged from a loss of 0.2 kg/yr to a gain of 1.1 kg/yr with an average gain of about 0.2 kg/yr in subjects not changing the PAL between baseline and follow-up, reflecting the fattening with increasing age corrected for the change in PAL.

In a laboratory study, Stubbs et al. showed that reducing the PAL from 1.8 to 1.4 over 7 days markedly affected energy balance. A change to a sedentary routine did not induce a compensatory reduction of energy intake and most of the excess energy was stored as fat [5]. Similarly, weight gain was observed in runners because of reductions in weekly exercise and was not reversed by resuming prior activity [6], showing that intake follows more an increase than a decrease in activity induced changes in daily energy expenditure. Here, a change from an active to a more sedentary life-style resulted in fat storage reflecting insufficient adaptation of food intake to a reduced energy requirement, even in the long term.
